# Mantis-like claw clip as a rescue therapy for the closure of an unexpected cricopharyngeal mucosal tear during peroral endoscopic myotomy

**DOI:** 10.1055/a-2368-4157

**Published:** 2024-08-07

**Authors:** Oscar V. Hernández Mondragón, Julio Gil Ferral Mejia

**Affiliations:** 1 Department of Endoscopy, Specialties Hospital, XXI Century National Medical Center, Mexico City, Mexico

The closure of an inadvertent mucosal defect during a third-space procedure is mandatory to avoid severe adverse events 1 . Multiple closure methods have been described (endoscopic clips, suture device, cyanoacrylate, over-the-scope [OTS] clip, etc.), with excellent outcomes 2 . However, some anatomic areas such as the cricopharynx can be difficult to close. A recently released mantis-like claw clip named Mantis clip (Boston Scientific, Marlborough, Massachusetts, USA) has shown excellent outcomes in the closure of gastrointestinal defects less than 30mm in size 3 and fixation of esophageal stents 4 , even in patients with a high risk of bleeding 5 .

A 55-year-old woman with type I achalasia, a history of Heller myotomy 18 years previously, and an Eckardt score of 9, underwent conventional peroral endoscopic myotomy. However, after closure of the entry site with an OTS clip (Ovesco Endoscopy AG, Tübingen, Germany), a large cricopharyngeal mucosal tear with oozing bleeding was observed ( Fig. 1 ). Multiple attempts at closure with conventional clips were performed without success. A t-type OTS clip was placed, achieving partial closure of the defect; however, a second OTS clip could not be placed ( Fig. 2 ). Finally, a first Mantis clip was used to achieve adequate approximation of the mucosal edges, then the closure was completed with a conventional clip and a final Mantis clip. No leakage was shown on the 24-hour water-soluble contrast study.

**Fig. 1:**
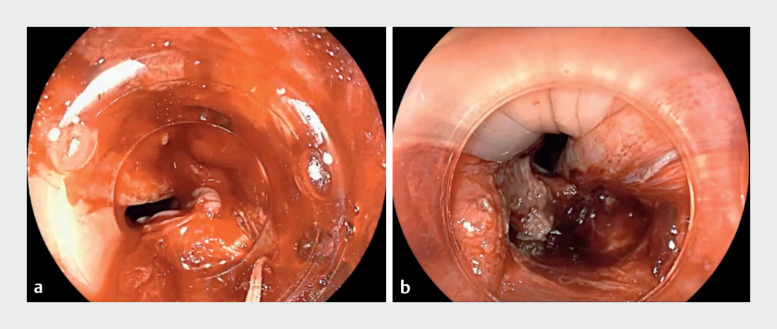
**a** Active bleeding in the cricopharyngeal area secondary to over-the-scope (OTS) clip placement during peroral endoscopic myotomy. **b** An unexpected large cricopharyngeal mucosal tear can be seen.

**Fig. 2:**
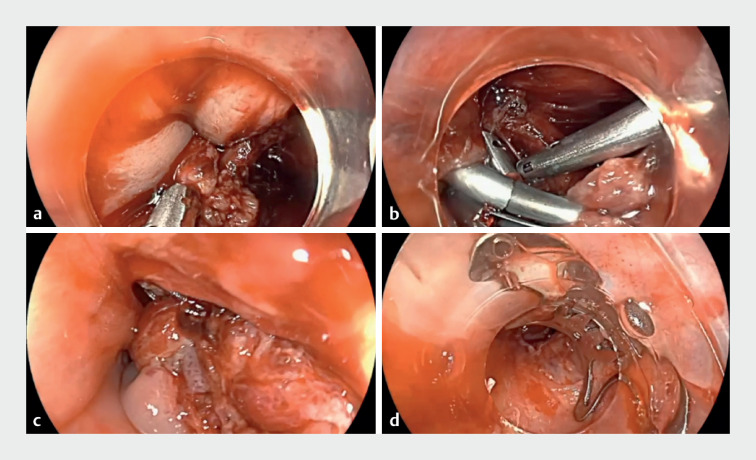
**a** Initially, conventional clips were used to attempt closure of this large tear. **b** Due to excessive wall tension, the clips became dislodged. **c** All clips were removed and an OTS clip was correctly placed at the distal end of the cricopharyngeal tear. **d** A persistent mucosal defect remain. A second OTS clip could not be placed in this difficult area.

The patient showed no clinical evidence of systemic inflammatory response. Liquids were initiated after 24 hours, and the diet was progressed to soft and then normal during the following week with no adverse events. Upper endoscopy after 2 months showed complete repair of the mucosal defect ( Fig. 3 , Video 1 ).

**Fig. 3:**
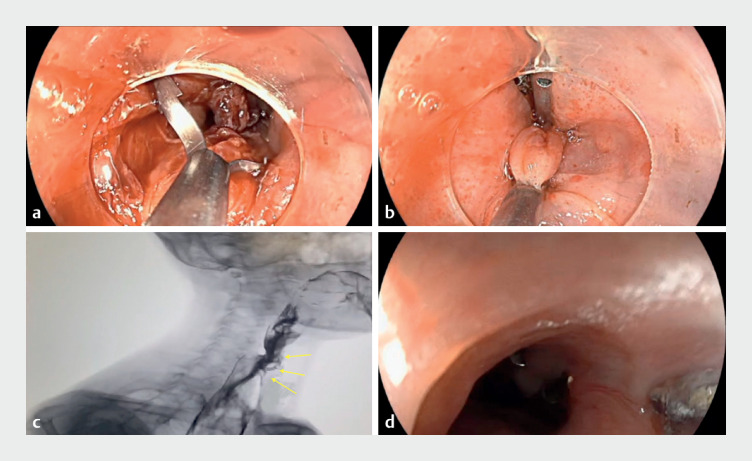
**a** A Mantis clip was used as a rescue therapy, with the advantage that it grasped the edges of the tear securely, avoiding slippage. **b** The mucosal tear was completely closed. **c** Water-soluble contrast study did not show any leakage. **d** Mucosal healing as observed 2 months later.

Mantis-like claw clip used as a rescue therapy for closure of an unexpected cricopharyngeal mucosal tear during peroral endoscopic myotomy.Video 1

In conclusion, the Mantis clip is an easy-to-use and safe alternative tool for the management of mucosal defects even in difficult areas such as the one shown in this case.

Endoscopy_UCTN_Code_CPL_1AH_2AJ

## Notes

Endoscopy E-Videos https://eref.thieme.de/e-videos
*E-Videos* is an open access online section of the journal *Endoscopy* , reporting on interesting cases and new techniques in gastroenterological endoscopy. All papers include a high-quality video and are published with a Creative Commons CC-BY license. Endoscopy E-Videos qualify for HINARI discounts and waivers and eligibility is automatically checked during the submission process. We grant 100% waivers to articles whose corresponding authors are based in Group A countries and 50% waivers to those who are based in Group B countries as classified by Research4Life (see: https://www.research4life.org/access/eligibility/ ). This section has its own submission website at https://mc.manuscriptcentral.com/e-videos .

## References

[1] Zhang XC Li QL Xu MD Major perioperative adverse events of peroral endoscopic myotomy: a systematic 5-year analysis Endoscopy 2016 48 967 978 10.1055/s-0042-110397 27448052

[2] Nabi Z Reddy DN Ramchandani M Adverse events during and after per-oral endoscopic myotomy: prevention, diagnosis, and management Gastrointest Endosc 2018 87 4 17 10.1016/j.gie.2017.09.029 28987545

[3] Nishiyama N Matsui T Nakatani K Novel strategy of hold-and-drag clip closure with mantis-like claw for post-gastric endoscopic submucosal dissection defect of <30 mm Endoscopy 2023 55 E1244 1245 38128588 10.1055/a-2213-4313 PMC10898239

[4] Kubota Y Nishiyama R Sasaki M Fixation of an esophageal stent using a novel re-openable endoclip for a tracheoesophageal fistula DEN open 2024 2 e342 10.1002/deo2.342 PMC10908370 38434147

[5] Inada T Sumida Y Homma H Novel clip method for endoscopic submucosal dissection defect closure reducing submucosal dead space in antithrombotic gastric patients Endoscopy 2024 56 E45 E46 10.1055/a-2223-4475 38232769 PMC10794086

